# The Geriatric Nutritional Risk Index Predicts Short‐ and Long‐Term Outcomes in the Oldest‐Old With Colorectal Cancer: A Multi‐Institutional Analysis of 225 Nonagenarians

**DOI:** 10.1002/ags3.70288

**Published:** 2026-09-29

**Authors:** Toshiaki Toshima, Fuminori Teraishi, Satoe Takanaga, Toshiharu Mitsuhashi, Ryo Inada, Tsuyoshi Ohtani, Ryosuke Yoshida, Naoto Hori, Kaoru Shigemitsu, Sumiharu Yamamoto, Tetsushi Kubota, Yuka Okano, Tetsuji Nobuhisa, Fumitaka Taniguchi, Wataru Ishikawa, Ryohei Shoji, Tatsuo Matsuda, Tatsuo Umeoka, Toshiyoshi Fujiwara, The Setouchi Colorectal Neoplasm Registration Study Group Collaborators

**Affiliations:** ^1^ Department of Surgery Kagawa Rosai Hospital Kagawa Japan; ^2^ Department of Gastroenterological Surgery Okayama University Hospital Okayama Japan; ^3^ Department of Surgery National Hospital Organization Fukuyama Medical Center Hiroshima Japan; ^4^ Center for Innovative Clinical Medicine, Medical Development Field Okayama University Okayama Japan; ^5^ Department of Surgery Kochi Health Sciences Center Kochi Japan; ^6^ Department of Surgery Saiseikai Okayama Hospital Okayama Japan; ^7^ Department of Surgery Okayama Rosai Hospital Okayama Japan; ^8^ Department of Surgery Tottori Municipal Hospital Tottori Japan; ^9^ Department of Surgery Tsuyama Chuo Hospital Okayama Japan; ^10^ Department of Surgery Okayama City Hospital Okayama Japan; ^11^ Department of Surgery Kobe Red Cross Hospital Kobe Hyogo Japan; ^12^ Department of Surgery Onomichi City Hospital Hiroshima Japan; ^13^ Department of Surgery Himeji Red Cross Hospital Himeji Hyogo Japan; ^14^ Department of Surgery National Hospital Organization Iwakuni Clinical Center Yamaguchi Japan; ^15^ Department of Surgery Fukuyama City Hospital Hiroshima Japan; ^16^ Department of Surgery Matsuda Hospital Okayama Japan; ^17^ Department of Surgery Matsuyama City Hospital Matsuyama Ehime Japan

**Keywords:** colorectal cancer, geriatric nutritional risk index, oldest‐old patients, overall survival, surgical outcome

## Abstract

**Aim:**

Surgical outcomes for colorectal cancer (CRC) in nonagenarians remain uncertain due to frailty, comorbidities, and malnutrition. The Geriatric Nutritional Risk Index (GNRI) is associated with surgical outcomes in older patients with CRC, but its prognostic value in nonagenarians is unclear. This study evaluated the GNRI's predictive value for short‐ and long‐term surgical outcomes in this population.

**Methods:**

We retrospectively analyzed 225 nonagenarians undergoing surgical resection of CRC at 15 institutions between 2011 and 2022. Patients were stratified by GNRI‐high/low status using two cutoffs: 98 (Cohort 1) and 86 (Cohort 2). Outcomes included postoperative complications, length of hospital stay, and overall survival (OS). Stabilized inverse‐probability‐of‐treatment weighting (IPTW) was used to adjust for measured confounding.

**Results:**

The median age was 92 years (female, 64.9%; colon cancer, 79.6%). GNRI‐high patients had higher body mass index and albumin levels. Complication rates did not differ between the groups. After adjustment, GNRI‐high patients in Cohort 1 had a shorter expected hospital stay (ratio 0.70, 95% CI 0.51–0.96; *p* = 0.025) but no significant OS difference. In Cohort 2, GNRI‐high patients had better OS (*p* < 0.01), confirmed by adjusted Cox models (HR 0.54, 95% CI 0.35–0.82; *p* < 0.01).

**Conclusions:**

Preoperative GNRI was associated with hospital stay and OS in nonagenarians undergoing CRC surgery, although the OS association was significant only with the cohort‐derived cutoff of 86, which requires external validation, and not with the literature‐based cutoff of 98. The GNRI is a simple tool to stratify surgical risk and optimize perioperative nutritional status in this vulnerable population.

## Graphical abstract

This multi‐institutional study evaluated the Geriatric Nutritional Risk Index (GNRI) in 225 nonagenarians undergoing surgical resection for colorectal cancer. A lower preoperative GNRI was independently associated with a longer hospital stay and poorer overall survival, even after adjustment for confounders using inverse‐probability‐of‐treatment weighting. These findings support incorporating GNRI‐based nutritional screening into preoperative risk assessment for the oldest‐old patients undergoing colorectal cancer surgery.

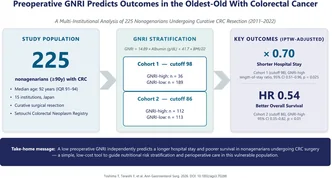

## 1 Introduction

The global demographic landscape is undergoing a significant shift towards an aging population, resulting in a substantial increase in the number of older individuals diagnosed with colorectal cancer (CRC) [1]. While the concept of “natural‐end cancer” has been discussed in the context of patients with advanced age [2], the role of surgery for CRC in the oldest‐old, specifically those aged ≥ 90 years (nonagenarians), remains a complex and evolving area of research [3, 4, 5]. Advanced age has traditionally been considered a contraindication to surgery due to concerns regarding increased perioperative morbidity and mortality [6]. However, improvements in surgical techniques, anesthetic management, and postoperative care have improved outcomes even in the oldest‐old [7, 8, 9]. Nevertheless, nonagenarians often present with multiple risk factors, including comorbidities, frailty, and nutritional deficiencies, which can adversely affect their postoperative course [3, 10]. This highlights the importance of identifying predictive markers of adverse events and long‐term survival in this specific subgroup of patients.

Nutritional status is a well‐established predictor of postoperative outcomes in patients undergoing surgery for CRC [11, 12, 13, 14, 15, 16]. Malnutrition is associated with an increased risk of surgical site infection, prolonged hospital stay, and readmission [10, 11, 12]. Among various nutritional assessment tools, the Geriatric Nutritional Risk Index (GNRI) has emerged as a practical and easily calculated marker for assessing the nutritional status of older patients [13, 14, 15, 16, 17, 18]. The GNRI has been validated as a prognostic marker in various surgical settings, including CRC surgery. Previous studies have demonstrated that a low preoperative GNRI is associated with a higher risk of postoperative complications and worse long‐term survival in older patients undergoing curative resection for CRC [13, 14, 15, 16]. The GNRI has been shown to have prognostic value in older patients undergoing neoadjuvant chemoradiotherapy for advanced rectal cancer, as well as in the context of metastatic CRC [17, 18]. These findings underscore the clinical relevance of the GNRI as a predictive tool.

Although these studies provide compelling evidence of the clinical value of the GNRI, there is a paucity of data specifically examining its prognostic utility in nonagenarians with CRC. Given the unique physiological and clinical challenges of this population, it is imperative to explore whether the GNRI can provide valuable information to guide surgical decision‐making and personalize perioperative care. The present study aims to address this critical knowledge gap by evaluating the impact of preoperative GNRI on postoperative short‐ and long‐term outcomes in nonagenarians undergoing surgical resection of CRC at multiple institutions. We hypothesize that a low preoperative GNRI will be independently associated with adverse postoperative outcomes and worse long‐term survival in this population. We aim to shed light on the clinical significance of the GNRI in the oldest‐old, thereby improving the management of this vulnerable patient population.

## 2 Methods

### 2.1 Study Design and Population

This retrospective cohort study was conducted in accordance with the Strengthening the Reporting of Observational Studies in Epidemiology (STROBE) guidelines. Data were retrieved from the Setouchi Colorectal Neoplasm Registration database and managed using the REDCap (Research Electronic Data Capture) tool hosted at Okayama University Hospital, as previously described [19]. The Setouchi Colorectal Neoplasm Registration database includes information on the oldest‐old patients aged ≥ 90 years from 15 Okayama University‐affiliated hospitals between January 2011 and December 2022. A total of 402 cases of CRC in the oldest‐old patients were identified. The inclusion criterion was patients with CRC undergoing primary tumor resection. Patients with no nutritional evaluation and/or missing data were excluded. In total, 225 patients were included in the study (Figure 1).

**FIGURE 1:**
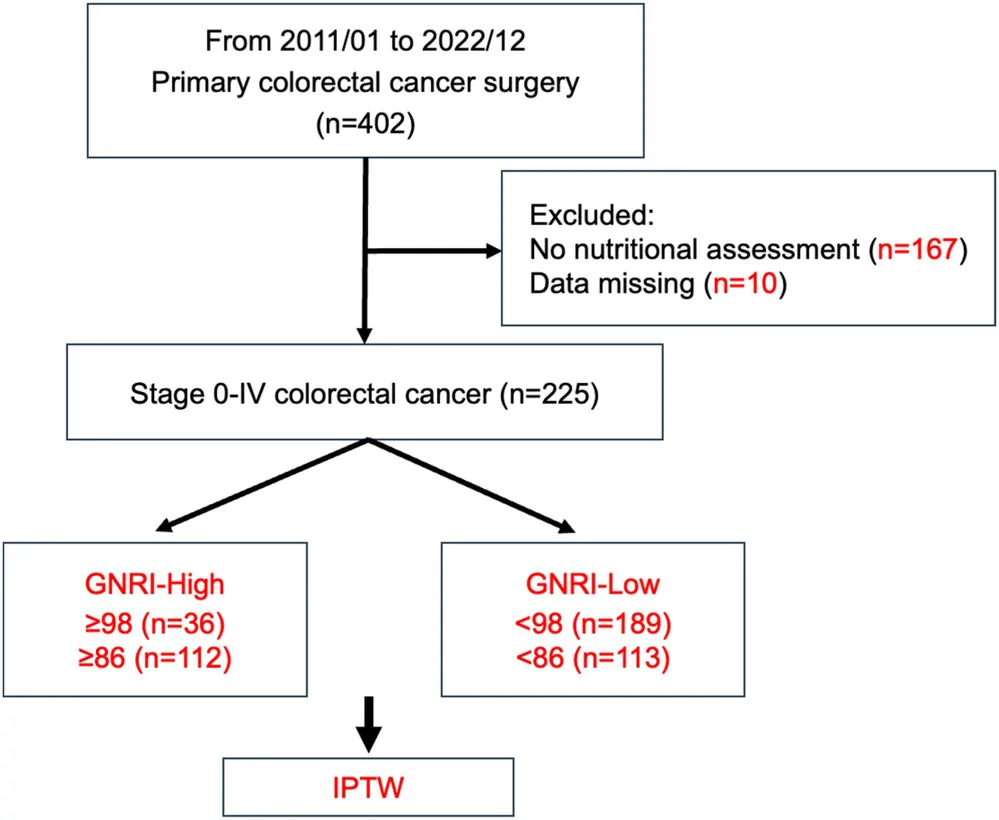
Patient selection flowchart. GNRI, Geriatric Nutritional Risk Index; IPTW, inverse‐probability‐of‐treatment weighting.

### 2.2 Geriatric Nutritional Risk Index (GNRI)

The baseline GNRI was calculated from serum albumin levels and body mass index (BMI) obtained on hospital admission, using the following formula: GNRI = 14.89 × serum albumin (g/dL) + 41.7 × BMI/22 [20]. Two cutoff values were evaluated. The literature‐based cutoff of 98 [13, 14, 15] was prespecified for the primary analysis. A data‐driven cutoff of 86, derived from a post hoc ROC analysis (empirical optimal cutoff, 85.99; rounded to 86), was used for the exploratory secondary analysis (Figure S1). Comparative analyses of short‐ and long‐term postoperative outcomes were performed using these cutoff values.

### 2.3 Outcomes

Postoperative complications were recorded for 30 days after surgery and graded according to the Clavien–Dindo classification [21]. Any deviation from normal postoperative status was considered a complication, and the treatment required to correct the complication was the basis for the grade of severity. Thus, complications that required medical therapy were classified as Grade II; complications that required surgical, endoscopic, or radiologic treatment as Grade III; life‐threatening complications as Grade IV; and postoperative death as Grade V. Complications classified as Grade II or higher were considered to be postoperative complications. The length of postoperative hospital stay was also assessed. Postoperative follow‐up was conducted according to the criteria of each participating hospital and the Colorectal Cancer Treatment Guidelines [22]. Overall survival (OS) was defined as the time interval from the date of surgery to the date of death.

### 2.4 Statistical Analysis

Continuous variables were presented as median (interquartile range [IQR]) and compared using the Mann–Whitney *U* test. Categorical variables were presented as numbers (percentages) and compared using Fisher's exact test or the chi‐square test, as appropriate. Baseline characteristics were also compared between the analyzed and excluded patients using the same statistical methods. Comparisons of continuous variables were based on available observations, and the number of missing observations was reported separately.

Postoperative complications were analyzed using modified Poisson regression with robust standard errors and reported as risk ratios (RRs) with 95% confidence intervals (CIs). Length of hospital stay was analyzed using negative‐binomial regression with robust standard errors and without an offset term. Exponentiated coefficients were reported as length‐of‐stay ratios (i.e., ratios of expected length of stay) with 95% CIs.

Propensity scores were estimated separately for each GNRI cutoff using logistic regression, with GNRI group as the dependent variable and age, sex, American Society of Anesthesiologists (ASA) classification, pathological stage (Stage ≤ II vs. ≥ III), surgical approach, and extent of lymph‐node dissection as covariates. For GNRI‐high patients, the stabilized inverse‐probability‐of‐treatment weight (IPTW) was calculated as the marginal probability of being GNRI‐high divided by the estimated propensity score. For GNRI‐low patients, it was calculated as the marginal probability of being GNRI‐low divided by one minus the estimated propensity score. These weights were applied to the modified Poisson regression model for postoperative complications, the negative‐binomial regression model for length of hospital stay, and the Cox proportional hazards model for OS. Patients with missing values in variables required for the propensity‐score model were excluded from the weighted analyses. The stability of the stabilized weights was evaluated by examining their distribution, range, and Kish effective sample size. The propensity score overlaps between the GNRI groups were assessed graphically. Covariate balance after weighting was evaluated using standardized mean differences, with an absolute standardized mean difference of less than 0.10 considered to indicate acceptable balance. Weight trimming or truncation was not applied [19, 23].

OS was estimated using the Kaplan–Meier method for both the unadjusted and stabilized IPTW‐weighted analyses. The survival curves were compared using the log‐rank test. Median survival time (MST) was estimated for each GNRI group in both analyses. Hazard ratios (HRs) and 95% CIs were estimated using unadjusted and stabilized IPTW‐weighted Cox proportional hazards models. As a sensitivity analysis for the primary GNRI cutoff of 98, the Cox proportional hazards analysis for OS was repeated after excluding patients with pathological Stage IV disease. The same stabilized IPTW approach and covariates as in the primary analysis were used.

A post hoc conventional ROC analysis was performed to determine a cohort‐specific GNRI cutoff. Patients who were alive at the last follow‐up but had less than 3 years of follow‐up were excluded because their 3‐year survival status could not be assessed. Among patients eligible for this analysis, vital status at the last follow‐up was used as the binary reference outcome. The AUC and its 95% confidence interval were calculated, and the optimal cutoff was estimated using the Liu method. Sensitivity and specificity at the optimal cutoff were also reported.

All statistical analyses were conducted using STATA/MP (version 18.0) (StataCorp, College Station, TX, USA). A probability “*p*” value less than 0.05 was considered statistically significant. Multiplicity was not considered because the study was exploratory.

## 3 Results

Among the 402 eligible patients, 177 were excluded because data required for GNRI calculation or propensity‐score weighting were unavailable. A comparison of the analyzed and excluded patients is presented in Table S1. The distributions of ASA classification (*p* = 0.001), history of abdominal surgery (*p* = 0.038), and pathological stage (*p* < 0.001) showed significant differences between the groups. The baseline characteristics of the patients are described in Table 1. The median (IQR) age was 92 (91–94) years (female, 66.7% [*n* = 150]; male, 33.3% [*n* = 75]). The primary tumor site was the colon in 180 patients (79.6%) and the rectum in 45 patients (20.4%). The median (IQR) GNRI was 85.9 (78.3–94.8). Patients were stratified into GNRI‐high and GNRI‐low groups according to two GNRI cutoff values: 98 (Cohort 1) (GNRI‐high, *n* = 36; GNRI‐low, *n* = 189) and 86 (Cohort 2) (GNRI‐high, *n* = 112; GNRI‐low, *n* = 113). A total of 124 patients were included in the ROC analysis. The AUC was 0.593 (95% CI 0.471–0.714). The Liu method identified an empirical optimal GNRI cutoff of 85.99, which was rounded to 86, with a sensitivity of 0.62 and specificity of 0.55 (Figure S1).

**TABLE 1:** Baseline patient characteristics before stabilized IPTW adjustment.

Variables	All (*n* = 225)	Cohort 1; GNRI cutoff value, 98		*p*	Cohort 2; GNRI cutoff value, 86		*p*
		GNRI‐high (*n* = 36)	GNRI‐low (*n* = 189)		GNRI‐high (*n* = 112)	GNRI‐low (*n* = 113)	
Age, years, median (IQR)	92 (91–94)	92 (91–94)	92 (91–94)	0.52	92 (91–94)	93 (91–95)	0.08
Gender, *n* (%)							
Male	75 (33.3)	16 (44.4)	59 (31.2)	0.12	42 (37.5)	33 (29.2)	0.19
Female	150 (66.7)	20 (55.6)	130 (68.8)		70 (62.5)	80 (70.8)	
BMI, kg/m^2^, median (IQR)	20.4 (18.2–22.5)	24.4 (22.4–26.2)	20.0 (17.8–21.5)	**< 0.001**	21.9 (20.0–23.8)	18.8 (16.8–20.7)	**< 0.001**
History of abdominal surgery, *n* (%)	45 (20.4)	9 (25)	36 (19.0)	0.41	21 (18.8)	24 (21.2)	0.64
Tumor location, *n* (%)							
Colon	180 (79.6)	28 (77.8)	152 (80.4)	0.72	87 (77.7)	93 (82.3)	0.39
Rectum	45 (20.4)	8 (22.2)	37 (19.6)		25 (22.3)	20 (17.7)	
Serum albumin, g/dL, median (IQR)	3.2 (2.8–3.7)	3.9 (3.7–4.1)	3.1 (2.7–3.5)	**< 0.001**	3.7 (3.4–3.9)	2.8 (2.4–3.0)	**< 0.001**
CEA, ng/mL, median (IQR)	4.9 (3.0–9.3)	4.4 (2.4–6.9)	5.1 (3.2–11.5)	0.056	5.4 (3.0–11.7)	4.6 (3.0–8.0)	0.33
CA19‐9, U/mL, median (IQR)	13.3 (5.6–28.0)	11.1 (6.1–19.0)	13.5 (5.3–33.6)	0.24	13.4 (6.5–31.8)	12.4 (5.0–27.9)	0.61
ASA classification, *n* (%)							
1	1 (0.4)	1 (2.8)	0	0.055	1 (0.9)	0	0.20
2	106 (47.1)	20 (55.6)	86 (45.5)		57 (50.9)	49 (43.4)	
3	112 (49.8)	15 (41.6)	97 (51.3)		53 (47.3)	59 (52.2)	
4	6 (2.7)	0	6 (3.2)		1 (0.9)	5 (4.4)	
GNRI score, median (IQR)	85.9 (78.3–94.8)	103.3 (100.0–107.2)	84.4 (75.4–90.1)	**< 0.001**	94.8 (89.7–100)	78.4 (70.2–83.2)	**< 0.001**
pStage, *n* (%)							
0	1 (0.4)	0	1 (0.5)	**0.03**	0	1 (0.9)	0.07
I	23 (10.2)	9 (25)	14 (7.4)		16 (14.3)	7 (6.2)	
II	98 (43.6)	14 (38.9)	84 (44.5)		45 (40.2)	53 (46.9)	
III	72 (32.0)	9 (25)	63 (33.3)		40 (35.7)	32 (28.3)	
IV	31 (13.8)	4 (11.1)	27 (14.3)		11 (9.8)	20 (17.7)	

*Note:* Bold values indicate statistically significant differences or associations, defined as *p* < 0.05.

Abbreviations: ASA, American Society of Anesthesiologists; BMI, body mass index; CA19‐9, carbohydrate antigen 19–9; CEA, carcinoembryonic antigen; GNRI, Geriatric Nutritional Risk Index; IPTW, inverse‐probability‐of‐treatment weight; IQR, interquartile range; pStage, pathological stage.

Age and sex distributions were comparable between the GNRI‐high and GNRI‐low groups in both cohorts. BMI and serum albumin levels were significantly higher in GNRI‐high patients than in GNRI‐low patients in both cohorts (both *p* < 0.001), whereas there were no significant differences in the history of abdominal surgery or tumor location between the GNRI‐high and GNRI‐low groups in either cohort. In Cohort 1, GNRI‐low patients tended to have higher carcinoembryonic antigen levels than GNRI‐high patients, although this difference did not reach statistical significance (*p* = 0.056), and CA19‐9 levels did not differ significantly between the groups (*p* = 0.24). Pathological stage distribution differed significantly between the GNRI‐high and GNRI‐low groups in Cohort 1 (*p* = 0.03), with a higher proportion of Stage III/IV disease in the GNRI‐low group, whereas this difference was not statistically significant in Cohort 2 (*p* = 0.07).

Table 2 presents the perioperative and surgical outcomes of the study cohort. Right‐sided colectomy was the most common procedure (103/225, 45.8%), followed by rectal surgery (48/225, 21.3%), left‐sided colectomy (39/225, 17.3%), and other procedures (35/225, 15.6%), with no significant difference in the distribution of surgical procedures between the GNRI‐high and GNRI‐low groups in either Cohort 1 (*p* = 0.44) or Cohort 2 (*p* = 0.60). Emergency surgery was performed in 20 patients (8.9%) overall, with no significant difference between groups in either cohort (Cohort 1: 5.6% vs. 9.5%, *p* = 0.44; Cohort 2: 6.3% vs. 11.5%, *p* = 0.17).

**TABLE 2:** Surgical outcomes before stabilized IPTW adjustment.

Variables	All (*n* = 225)	Cohort 1; GNRI cutoff value, 98		*p*	Cohort 2; GNRI cutoff value, 86		*p*
		GNRI‐high (*n* = 36)	GNRI‐low (*n* = 189)		GNRI‐high (*n* = 112)	GNRI‐low (*n* = 113)	
Surgical approach, *n* (%)							
Open	88 (39.1)	8 (22.2)	80 (42.3)	**0.02**	33 (29.5)	55 (48.7)	**< 0.01**
Laparoscopy	137 (60.9)	28 (77.8)	109 (57.7)		79 (70.5)	58 (51.3)	
Surgical procedure, *n* (%)							
Right‐sided colectomy	103 (45.8)	14 (38.9)	89 (47.1)	0.44	48 (42.9)	55 (48.7)	0.60
Left‐sided colectomy	39 (17.3)	9 (25)	30 (15.9)		19 (17)	20 (17.7)	
Rectal surgery	48 (21.3)	9 (25)	39 (20.6)		28 (25)	20 (17.7)	
Others	35 (15.6)	4 (11.1)	31 (16.4)		17 (15.1)	18 (15.9)	
Lymph node dissection, *n* (%)							
D3	141 (62.7)	24 (66.7)	117 (61.9)	0.59	74 (66.1)	67 (59.3)	0.29
Non‐D3	84 (37.3)	12 (33.3)	72 (38.1)		38 (33.9)	46 (40.7)	
Emergency surgery, *n* (%)	20 (8.9)	2 (5.6)	18 (9.5)	0.44	7 (6.3)	13 (11.5)	0.17
Stoma creation, *n* (%)	37 (16.4)	0	37 (19.6)	**< 0.01**	8 (7.1)	29 (25.7)	**< 0.001**
R0 resection, *n* (%)	193 (85.8)	32 (88.9)	161 (85.2)	0.56	101 (90.2)	92 (81.4)	0.06
Operation time, min, median (IQR)	171 (121–233)	219 (163–264)	164 (121–223)	**< 0.01**	194 (133–252)	154 (118–211)	**< 0.01**
Blood loss, ml, median (IQR)	30 (5–100)	20 (5–145)	30 (5–100)	0.99	20 (0–75)	48 (10–132)	**0.02**
Post operative complications, *n* (%)							
C‐D Grade I‐V	82 (36.4)	14 (38.9)	68 (36.0)	0.74	42 (37.5)	40 (35.4)	0.74
C‐D Grade III‐V	13 (5.8)	2 (5.6)	11 (5.8)	0.95	8 (7.1)	5 (4.4)	0.38
Surgical site infections	3 (1.4)	0	3 (1.6)		1 (0.9)	2 (1.8)	
Anastomotic leakage	2 (0.9)	1 (2.8)	1 (0.5)		2 (1.8)	0	
Pneumonia	3 (1.4)	0	3 (1.6)		2 (1.8)	1 (0.9)	
Cardiovascular events	1 (0.4)	0	1 (0.5)		0	1 (0.9)	
Others	4 (1.7)	1 (2.8)	3 (1.6)		3 (2.7)	1 (0.9)	
30‐day mortality, *n* (%)	2 (0.9)	0	2 (1.0)	NA	0	2	NA
Length of stay, days, median (range)	16 (11–23)	14 (10–22)	16 (12–24)	0.09	15 (11–26)	16 (13–23)	0.15
Reoperation, *n* (%)	1 (0.4)	0	1 (0.5)	NA	0	1 (0.9)	NA

*Note:* Bold values indicate statistically significant differences or associations, defined as *p* < 0.05.

Abbreviations: C‐D, Clavien–Dindo; GNRI, Geriatric Nutritional Risk Index; IPTW, inverse‐probability‐of‐treatment weight; IQR, interquartile range; R0, residual tumor 0.

In both cohorts, GNRI‐high patients underwent laparoscopic surgery more frequently and had longer operative times than GNRI‐low patients (Cohort 1: *p* = 0.02 and *p* < 0.01, respectively; Cohort 2: *p* < 0.01 and *p* < 0.01, respectively). Stoma creation was significantly less frequent in GNRI‐high patients (Cohort 1: 0% vs. 19.6%, *p* < 0.01; Cohort 2: 7.1% vs. 25.7%, *p* < 0.001). There was no significant difference in the R0 resection rate between groups in either cohort (Cohort 1: 88.9% vs. 85.2%, *p* = 0.56; Cohort 2: 90.2% vs. 81.4%, *p* = 0.06). In Cohort 1, GNRI‐high patients tended to have shorter hospital stays (*p* = 0.09), although this difference was not statistically significant. In Cohort 2, GNRI‐high patients had significantly less blood loss (*p* = 0.02).

Postoperative complications of Clavien‐Dindo Grade I–V occurred in 82 patients (36.4%), and Grade III–V complications occurred in 13 patients (5.8%), with no significant difference between the GNRI‐high and GNRI‐low groups in either cohort (Cohort 1: 38.9% vs. 36.0%, *p* = 0.74, and 5.6% vs. 5.8%, *p* = 0.95; Cohort 2: 37.5% vs. 35.4%, *p* = 0.74, and 7.1% vs. 4.4%, *p* = 0.38). Among the specific types of severe complications (Grade III–V), surgical site infection occurred in 3 patients (1.4%), anastomotic leakage in 2 (0.9%), pneumonia in 3 (1.4%), cardiovascular events in 1 (0.4%), and other complications in 4 (1.7%). In Cohort 1, these occurred more frequently in the GNRI‐low group than in the GNRI‐high group (surgical site infection: 0 vs. 3 [1.6%]; anastomotic leakage: 1 [2.8%] vs. 1 [0.5%]; pneumonia: 0 vs. 3 [1.6%]; cardiovascular events: 0 vs. 1 [0.5%]), whereas in Cohort 2, the distribution was more balanced (surgical site infection: 1 [0.9%] vs. 2 [1.8%]; anastomotic leakage: 2 [1.8%] vs. 0; pneumonia: 2 [1.8%] vs. 1 [0.9%]; cardiovascular events: 0 vs. 1 [0.9%]). Because event numbers for each specific complication were small, formal statistical comparisons between GNRI groups were not performed for these individual complications. Thirty‐day mortality occurred in 2 patients (0.9%) and reoperation was required in 1 patient (0.4%), all in the GNRI‐low group.

Stabilized IPTW balanced baseline characteristics (Table S2). For the GNRI cutoffs of 98 and 86, the mean stabilized weights were 1.004 and 0.998, respectively, and the corresponding ranges were 0.373–3.514 and 0.585–2.432. The overall Kish effective sample sizes were 206.2 and 207.0, corresponding to 91.6% and 92.0% of the original sample size, respectively. All absolute standardized mean differences after weighting were below 0.10. The stabilized‐weight histograms did not indicate severely extreme weights, and the propensity‐score distributions showed substantial overlap between the GNRI groups (Figure S2).

Unadjusted risk ratios (RRs) for postoperative complications did not differ significantly between the GNRI‐high and GNRI‐low groups at either cutoff (cutoff 98: RR 1.07, 95% CI 0.68–1.67; cutoff 86: RR 1.08, 95% CI 0.77–1.53) (Table 3a). Stabilized IPTW‐weighted modified Poisson analyses likewise showed no significant between‐group differences in postoperative complications. For length of hospital stay, the stabilized IPTW‐weighted length‐of‐stay ratio was significantly lower in the GNRI‐high group at the cutoff of 98 (ratio 0.70, 95% CI 0.51–0.96; *p* = 0.025), but not at the cutoff of 86 (Table 3b).

**TABLE 3:** (a) Risk ratio for postoperative complications and (b) length‐of‐stay ratio in GNRI‐high patients compared to GNRI‐low patients.

GNRI cutoff value	Crude analysis model	*p*	Adjusted model	*p*
	Odds ratio (95% CI)		Odds ratio (95% CI)	
(a)				
≥ 98 (reference: < 98)	1.07 (0.68, 1.67)	0.784	0.92 (0.52, 1.64)	0.771
≥ 86 (reference: < 86)	1.08 (0.77, 1.53)	0.643	1.07 (0.75, 1.52)	0.702
(b)				
≥ 98 (reference: < 98)	0.76 (0.54, 1.06)	0.102	**0.70 (0.51, 0.96)**	**0.025**
≥ 86 (reference: < 86)	0.90 (0.62, 1.31)	0.582	1.00 (0.71, 1.40)	0.999

*Note:* Bold values indicate statistically significant differences or associations, defined as *p* < 0.05.

Abbreviations: CI, confidence interval; GNRI, Geriatric Nutritional Risk Index.

For the Cohort 1 (cutoff of 98), the unadjusted Kaplan–Meier analysis showed no significant difference in OS between the GNRI groups (*p* = 0.125; Figure 2a), and the stabilized IPTW‐weighted analysis yielded a similar result (*p* = 0.156; Figure 2b). For the Cohort 2 (cutoff of 86), the GNRI‐high group had significantly longer OS in both the unadjusted analysis (*p* = 0.002; Figure 3a) and the stabilized IPTW‐weighted analysis (*p* = 0.004; Figure 3b). In the stabilized IPTW‐weighted Cox models, the HR was 0.67 (95% CI 0.39–1.16; *p* = 0.156) for the Cohort 1 (cutoff of 98) and 0.54 (95% CI 0.35–0.82; *p* = 0.004) for the Cohort 2 (cutoff of 86) (Table 4). The MSTs for the GNRI‐high versus GNRI‐low groups were 1206 versus 924 days for the cutoff of 98 and 1283 versus 700 days for the cutoff of 86; these median estimates were unchanged after weighting (Table S3). In the sensitivity analysis excluding patients with pathological Stage IV disease, 194 patients remained. For the GNRI‐high group (≥ 98) versus the GNRI‐low group (< 98), the unadjusted HR for death was 0.57 (95% CI 0.29–1.15; *p* = 0.117), and the stabilized IPTW‐adjusted HR was 0.52 (95% CI 0.27–0.98; *p* = 0.044). There was no evidence of violation of the proportional hazards assumption in the unadjusted or adjusted model (*p* = 0.144 and *p* = 0.165, respectively). During follow‐up, 100 of the 225 patients died. Of these deaths, 18 (18%) were attributed to the index CRC, 33 (33%) to other causes, and 48 (48%) had an undetermined cause of death. All 18 confirmed CRC‐related deaths occurred in the GNRI‐low group in Cohort 1 (GNRI < 98), whereas no confirmed CRC‐related deaths occurred in the GNRI‐high group (GNRI ≥ 98).

**FIGURE 2:**
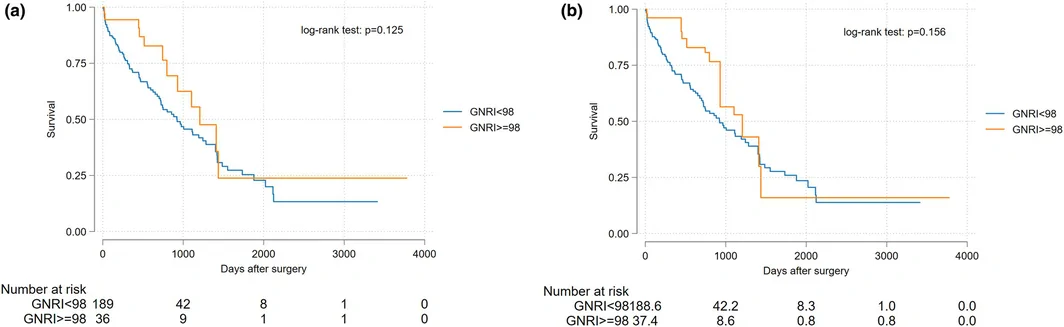
Overall survival according to the GNRI cutoff of 98. (a) Unadjusted Kaplan–Meier curves of OS according to high and low GNRI. (b) Stabilized IPTW‐weighted Kaplan–Meier curves of OS according to high and low GNRI (log‐rank test, *p* = 0.13). CRC, colorectal cancer; GNRI, Geriatric Nutritional Risk Index; IPTW, inverse‐probability‐of‐treatment weighting; OS, overall survival.

**FIGURE 3:**
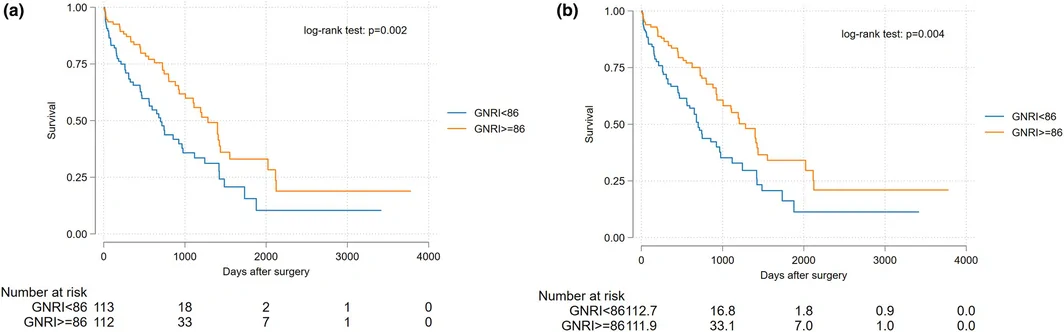
Overall survival according to the GNRI cutoff of 86. (a) Unadjusted Kaplan–Meier curves of OS according to high and low GNRI. (b) Stabilized IPTW‐weighted Kaplan–Meier curves of OS according to high and low GNRI (log‐rank test, *p* = 0.004). CRC, colorectal cancer; GNRI, Geriatric Nutritional Risk Index; IPTW, inverse‐probability‐of‐treatment weighting; OS, overall survival.

**TABLE 4:** Cox proportional hazard ratios for overall survival according to GNRI cutoff values, before and after IPTW adjustment.

	*N*	Hazard ratio (95% CI)	*p*	Proportional hazard test
Cohort 1				
GNRI cutoff value, 98				
Crude model	225	0.63 (0.34, 1.14)	0.129	0.138
Adjusted model	225	0.67 (0.39, 1.16)	0.156	0.126
Cohort 2				
GNRI cutoff value, 86				
Crude model	225	**0.53 (0.36, 0.80)**	**0.002**	0.227
Adjusted model	225	**0.54 (0.35, 0.82)**	**0.004**	0.337

*Note:* Bold values indicate statistically significant differences or associations, defined as *p* < 0.05.

Abbreviations: GNRI, Geriatric Nutritional Risk Index; IPTW, inverse‐probability‐of‐treatment weighting.

## 4 Discussion

This multi‐institutional study evaluated the predictive value of the GNRI for postoperative outcomes and OS in a large cohort of 225 nonagenarians undergoing surgical resection of CRC. Our results demonstrate that a lower preoperative GNRI is associated with a longer expected length of hospital stay and poorer OS in this patient population. These findings underscore the importance of preoperative nutritional assessment and optimization in nonagenarians undergoing surgery for CRC.

This study builds on a growing body of literature that highlights the vulnerability of older patients with CRC, especially the oldest‐old, to adverse surgical outcomes. While the benefits of surgery for CRC in older patients are well established [6, 8, 9], nonagenarians often present with more complex clinical scenarios due to multiple comorbidities, reduced physiological reserve, and a higher prevalence of frailty [3, 4, 5, 24]. These factors contribute to their increased susceptibility to postoperative complications and mortality, necessitating the development of robust risk stratification strategies [25]. This study provides crucial evidence that preoperative nutritional assessment with the GNRI can serve as an independent predictor of patient outcomes.

Our results are consistent with those of previous studies examining the association between GNRI and the outcomes of older patients with CRC [13, 14, 15, 16]. A meta‐analysis showed that a lower GNRI was associated with higher rates of postoperative complications and poorer long‐term survival in older patients with CRC [26, 27]. This underscores the importance of the GNRI as a practical and readily available tool for identifying high‐risk patients [28]. The prognostic utility of the GNRI has also been demonstrated in patients receiving neoadjuvant therapy for rectal cancer and in patients with metastatic disease [17, 18], expanding its role in the management of older patients with CRC. The data‐driven cutoff of 86 was derived from the present cohort and showed modest discriminatory ability for vital status. Therefore, this cutoff should be regarded as exploratory and requires external validation. Accordingly, the literature‐based cutoff of 98 was retained for the primary analysis.

Several mechanisms may explain the association between a low GNRI and adverse postoperative outcomes. Firstly, a low GNRI indicates a poor nutritional status, which can lead to impaired wound healing, reduced immune function, and increased susceptibility to infection [29]. These factors increase the risk of postoperative complications, such as surgical site infection, anastomotic leakage, and pneumonia [30]. Secondly, malnutrition can lead to muscle wasting, reduced functional capacity, and frailty [31], further compromising the ability of nonagenarians to recover from surgery. Thirdly, patients with a low GNRI may have systemic inflammation, which has been shown to be associated with adverse surgical outcomes [11]. Our findings emphasize the importance of incorporating nutritional assessment into preoperative risk stratification and the need to prioritize nutritional support in high‐risk patients.

This study highlights the importance of the GNRI in predicting long‐term survival in nonagenarians with CRC. A previous study has shown that preoperative malnutrition is associated with a poorer prognosis in older patients with various cancers, including CRC [31]. Our results confirmed that patients with a low GNRI at the time of surgery had worse OS than those with a high GNRI. In the sensitivity analysis excluding patients with pathological Stage IV disease, the association was directionally consistent with the primary analysis and became statistically significant after IPTW adjustment. This finding suggests that the association between a higher GNRI and lower mortality was not attributable solely to the inclusion of patients with metastatic disease. However, this post hoc sensitivity analysis should be interpreted cautiously. This suggests that the impact of malnutrition on survival is not limited to the perioperative period. The association between a low GNRI and poor survival may be explained by several factors, including an increased risk of recurrence, reduced response to adjuvant therapy, and increased susceptibility to infectious complications [29]. Therefore, preoperative nutritional optimization may impact not only perioperative outcomes but also long‐term outcomes in this vulnerable population.

While this study provides compelling evidence supporting the clinical significance of the GNRI, it is important to acknowledge its limitations. First, the retrospective design and complete‐case analysis may have introduced selection bias. Of the 402 eligible patients, 177 were excluded. Compared with the analyzed patients, the excluded patients showed different distributions of ASA classification, history of abdominal surgery, and pathological stage (Table S1). Therefore, the analyzed cohort may not be representative of the entire source population. Second, the GNRI is a simple tool that does not capture all aspects of nutritional status, including specific micronutrient deficiencies and inflammatory markers. The inclusion of other nutritional assessment tools, such as the Subjective Global Assessment [12] or the Comprehensive Geriatric Assessment [32], in future studies may provide a more detailed assessment of nutritional status. Third, while we adjusted for several confounding factors, residual confounding may persist, and the observed association between the GNRI and patient outcomes may be affected by other unmeasured variables. Fourth, the cause of death could not be reliably determined in 48 of the 100 deaths (48%). This largely reflects the practical challenges of post‐discharge follow‐up, as some patients were followed by primary care physicians while others relocated to live with family or were admitted to nursing facilities, limiting the availability of detailed information on recurrence and cause of death. Moreover, all 18 confirmed CRC‐related deaths occurred in patients with a GNRI < 98, whereas no CRC‐related deaths were observed in those with a GNRI ≥ 98, resulting in a zero‐event stratum. Given the substantial proportion of deaths with an undetermined cause and the resulting instability of effect estimates, cancer‐specific survival could not be evaluated reliably. Finally, this study included only patients who underwent surgical resection, and the possibility of selection bias cannot be excluded. The criteria for surgical indication were not standardized across institutions; at each center, the surgical approach and procedure were determined individually through preoperative multidisciplinary conferences, and the availability of colorectal surgical specialists also varied among institutions. This heterogeneity in decision‐making and surgeon expertise should be considered when interpreting the results. The rate of laparoscopic surgery was significantly higher in the GNRI‐high groups. This likely reflects clinical selection, as patients with lower GNRI tend to have greater frailty, more comorbidities, and more complex disease, leading surgeons to favor open surgery for safety. Institutional differences in the availability of colorectal surgical specialists may have further contributed to this selection. Given the greater invasiveness of open surgery, this imbalance likely confounded short‐term outcomes such as blood loss and length of stay; although IPTW was used to adjust for this in the survival analysis, residual confounding cannot be entirely excluded for these short‐term outcomes.

Despite these limitations, our study has several strengths. First, the use of a multi‐institutional database allowed us to examine a large cohort of nonagenarians undergoing surgery for CRC, enhancing the generalizability of our findings. Second, we used multivariable analysis to adjust for potential confounders, thereby providing a more robust assessment of the association between the GNRI and patient outcomes. Finally, we not only evaluated the impact of the GNRI on short‐term postoperative outcomes but also on long‐term survival, providing a more comprehensive picture of the impact of nutritional status on the surgical trajectory.

The implications of our findings for clinical practice are significant. First, we recommend that all nonagenarians undergoing surgery for CRC undergo preoperative nutritional screening using the GNRI. Second, patients with a low GNRI should be considered at high risk of adverse outcomes and referred for preoperative nutritional optimization, which may include dietary counseling, nutritional supplementation, and other interventions aimed at improving patients' nutritional status. Further research is needed to examine the impact of preoperative nutritional interventions on surgical outcomes in nonagenarians and to explore strategies for improving the nutritional status of patients with a low GNRI after surgery. Given the growing number of older patients undergoing cancer surgery, more resources are necessary to enhance preoperative nutritional assessment and management in this vulnerable population.

## 5 Conclusions

The GNRI is a simple and valuable tool for predicting postoperative outcomes in nonagenarians undergoing surgical resection of CRC. Preoperative GNRI is associated with a longer expected hospital stay and poorer long‐term survival, although the association with long‐term survival was observed only using a cohort‐derived cutoff of 86 and was not significant using the literature‐based cutoff of 98; therefore, this cutoff should be regarded as exploratory and requires validation in an independent cohort. Incorporating the GNRI into the preoperative risk assessment, followed by targeted nutritional interventions, may improve the outcomes of nonagenarians with CRC. Further research is warranted to examine strategies for improving the outcomes of nonagenarians undergoing surgery for CRC.

## Author Contributions

**Toshiaki Toshima:** conceptualization, methodology, investigation, data curation, writing – original draft. **Fuminori Teraishi:** conceptualization, methodology, investigation, data curation. **Satoe Takanaga:** methodology, data curation. **Toshiharu Mitsuhashi:** methodology, formal analysis. **Ryo Inada:** data curation, investigation. **Tsuyoshi Ohtani:** investigation, data curation. **Ryosuke Yoshida:** investigation, data curation. **Naoto Hori:** investigation, data curation. **Kaoru Shigemitsu:** investigation, data curation. **Sumiharu Yamamoto:** investigation, data curation. **Tetsushi Kubota:** investigation, data curation. **Yuka Okano:** investigation, data curation. **Tetsuji Nobuhisa:** investigation, data curation. **Fumitaka Taniguchi:** data curation, investigation. **Wataru Ishikawa:** investigation, data curation. **Ryohei Shoji:** investigation, data curation. **Tatsuo Matsuda:** data curation, investigation. **Tatsuo Umeoka:** investigation, data curation. **Toshiyoshi Fujiwara:** conceptualization, supervision.

## Funding

This study was supported by the Okayama Health Promotion Foundation for Cancer Research Grants (Okayama, Japan) and JSPS KAKENHI Grant Number JP25K12003.

## Ethics Statement

All procedures performed in studies involving human participants were in accordance with the ethical standards of the institutional and/or national research committee and with the 1964 Declaration of Helsinki and its later amendments or comparable ethical standards. The Institutional Review Board of Okayama University Hospital (approval number 2112‐036) and all participating hospitals approved this study as exempt human subject research.

## Conflicts of Interest

The authors declare no conflicts of interest.

## Supporting information


**Figure S1:** Receiver operating characteristic (ROC) curve for the GNRI in predicting 3‐year survival. The area under the curve was 0.593 (95% CI, 0.471–0.714). The empirical optimal cutoff determined using the Liu method was 85.99, rounded to 86, with a sensitivity of 0.62 and specificity of 0.55. AUC, area under the curve; CI, confidence interval; GNRI, Geriatric Nutritional Risk Index.
**Figure S2:** Propensity‐score distributions according to GNRI group after stabilized inverse‐probability‐of‐treatment weighting (IPTW). GNRI, Geriatric Nutritional Risk Index; IPTW, inverse‐probability‐of‐treatment weighting.

**Table S1:** Comparison of baseline characteristics between analyzed and excluded patients.
**Table S2:** Baseline characteristics after stabilized IPTW adjustment.
**Table S3a:** Median survival time analysis (unadjusted).
**Table S3b:** Median survival time analysis (after stabilized IPTW adjustment).

## Data Availability

The data that support the findings of this study are available on request from the corresponding author. The data are not publicly available due to privacy or ethical restrictions.
