# Supplementary material for: The Geriatric Nutritional Risk Index Predicts Short‐ and Long‐Term Outcomes in the Oldest‐Old With Colorectal Cancer: A Multi‐Institutional Analysis of 225 Nonagenarians
Source: Ann Gastroenterol Surg. 2026 Sep 29:10.1002/ags3.70288. Online ahead of print. doi: 10.1002/ags3.70288 (PMC13621199; doi:10.1002/ags3.70288)
Supplement: Supplementary file 1 [file AGS3-9999-0-s001.docx]

**Supplementary Table 1**	Comparison of baseline characteristics between analyzed and excluded patients

|  | All (n=402) | Excluded (n=177) | Analyzed (n=225) | p value |
| --- | --- | --- | --- | --- |
| Age, years, median (IQR) | 92.0 (91.0-94.0) | 92.0 (91.0-94.0) | 92.0 (91.0-94.0) | 0.059 |
| Sex, n (%) |  |  |  |  |
| female | 268 (66.7%) | 118 (66.7%) | 150 (66.7%) | 1.000 |
| male | 134 (33.3%) | 59 (33.3%) | 75 (33.3%) |  |
| BMI, kg/m^2, median (IQR) | 20.2 (18.1-22.4) | 20.0 (18.1-22.2) | 20.4 (18.2-22.5) | 0.539 |
| Missing, n | 10 | 10 | 0 |  |
| History of abdominal surgery, n (%) |  |  |  |  |
| no | 308 (76.6%) | 128 (72.3%) | 180 (80.0%) | 0.038 |
| yes | 91 (22.6%) | 46 (26.0%) | 45 (20.4%) |  |
| missing | 3 (0.7%) | 3 (1.7%) | 0 (0.0%) |  |
| Tumor location, n (%) |  |  |  |  |
| Colon | 320 (79.4%) | 140 (79.1%) | 180 (79.6%) | 0.294 |
| Rectum | 78 (19.4%) | 33 (18.6%) | 45 (20.4%) |  |
| missing | 4 (1.2%) | 4 (2.3%) | 0 (0.0%) |  |
| Serum albumin, g/dL, median (IQR) | 3.2 (2.7-3.6) | 3.2 (2.5-3.4) | 3.2 (2.8-3.7) | 0.294 |
| Missing, n | 160 | 160 | 0 |  |
| CEA, ng/mL, median (IQR) | 5.0 (3.0-9.5) | 7.3 (2.3-9.6) | 4.9 (3.0-9.3) | 0.406 |
| Missing, n | 170 | 159 | 11 |  |
| CA19-9, U/mL, median (IQR) | 13.1 (5.4-29.6) | 9.9 (4.4-42.1) | 13.3 (5.6-28.0) | 0.477 |
| Missing, n | 174 | 160 | 14 |  |
| ASA classification, n (%) |  |  |  |  |
| 1 | 2 (0.5%) | 1 (0.6%) | 1 (0.4%) | 0.001 |
| 2 | 166 (41.3%) | 60 (33.9%) | 106 (47.1%) |  |
| 3 | 220 (54.7%) | 108 (61.0%) | 112 (49.8%) |  |
| 4 | 8 (2.0%) | 2 (1.1%) | 6 (2.7%) |  |
| missing | 6 (1.5%) | 6 (3.4%) | 0 (0.0%) |  |
| GNRI score, median (IQR) | 85.9 (78.4-94.8) |  | 85.9 (78.3-94.8) |  |
| Missing, n | 177 | 177 | 0 |  |
| pStage, n (%) |  |  |  |  |
| 0 | 4 (1.0%) | 3 (1.7%) | 1 (0.4%) | < 0.001 |
| I | 43 (10.7%) | 20 (11.3%) | 23 (10.2%) |  |
| II | 172 (42.8%) | 73 (41.2%) | 98 (43.6%) |  |
| III | 114 (28.4%) | 43 (24.3%) | 72 (32.0%) |  |
| IV | 52 (12.9%) | 21 (11.9%) | 31 (13.8%) |  |
| Unknown | 17 (4.2%) | 17 (9.6%) | 0 (0.0%) |  |

Abbreviations: ASA, American Society of Anesthesiologists; BMI, body mass index; CA19-9, carbohydrate antigen 19-9; CEA, carcinoembryonic antigen; GNRI, Geriatric Nutritional Risk Index; pStage, pathological stage.

**Supplementary Table 2**	Baseline characteristics after stabilized IPTW adjustment

|  | **Crude analysis model** | | | | | |  | **Adjusted model by IPTW** | | | | | |
| --- | --- | --- | --- | --- | --- | --- | --- | --- | --- | --- | --- | --- | --- |
|  | **Above cutoff value** | | **Below cutoff value** | |  |  |  | **Above cutoff value** | | **Below cutoff value** | |  |  |
|  | mean | variance | mean | variance | Std.diff | Var.Ratio |  | mean | variance | mean | variance | Std.diff | Var.Ratio |
|  |  |  |  |  |  |  |  |  |  |  |  |  |  |
| Cohort 1 |  |  |  |  |  |  |  |  |  |  |  |  |  |
| GNRI cutoff value, 98 |  |  |  |  |  |  |  |  |  |  |  |  |  |
| Age | 92.69 | 4.56 | 93.13 | 7.20 | 0.18 | 0.63 |  | 93.23 | 5.13 | 93.08 | 7.00 | 0.06 | 0.73 |
| Gender | 0.44 | 0.25 | 0.31 | 0.22 | 0.27 | 1.18 |  | 0.32 | 0.22 | 0.33 | 0.22 | 0.03 | 1.00 |
| ASA classification | 2.39 | 0.30 | 2.58 | 0.31 | 0.34 | 0.98 |  | 2.55 | 0.30 | 2.55 | 0.31 | 0.01 | 0.98 |
| pStage | 0.36 | 0.24 | 0.47 | 0.25 | 0.22 | 0.95 |  | 0.47 | 0.26 | 0.45 | 0.25 | 0.03 | 1.03 |
| Surgical approach | 0.78 | 0.18 | 0.58 | 0.25 | 0.44 | 0.72 |  | 0.57 | 0.25 | 0.61 | 0.24 | 0.07 | 1.05 |
| Lymphnode dissection | 0.33 | 0.23 | 0.39 | 0.24 | 0.11 | 0.96 |  | 0.33 | 0.23 | 0.37 | 0.24 | 0.10 | 0.96 |
| Average value of Std.diff, Var.Ratio |  |  |  |  | 0.26 | 0.90 |  |  |  |  |  | 0.05 | 0.96 |
|  |  |  |  |  |  |  |  |  |  |  |  |  |  |
| Cohort 2 |  |  |  |  |  |  |  |  |  |  |  |  |  |
| GNRI cutoff value, 86 |  |  |  |  |  |  |  |  |  |  |  |  |  |
| Age | 92.71 | 5.97 | 93.41 | 7.40 | 0.27 | 0.81 |  | 93.09 | 7.56 | 93.11 | 6.72 | 0.01 | 1.12 |
| Gender | 0.38 | 0.24 | 0.29 | 0.21 | 0.18 | 1.13 |  | 0.33 | 0.22 | 0.32 | 0.22 | 0.02 | 1.02 |
| ASA classification | 2.48 | 0.29 | 2.61 | 0.33 | 0.23 | 0.87 |  | 2.54 | 0.31 | 2.54 | 0.32 | 0.00 | 0.98 |
| pStage | 0.45 | 0.25 | 0.46 | 0.25 | 0.03 | 0.99 |  | 0.47 | 0.25 | 0.47 | 0.25 | 0.00 | 1.00 |
| Surgical approach | 0.71 | 0.21 | 0.51 | 0.25 | 0.40 | 0.83 |  | 0.61 | 0.24 | 0.61 | 0.24 | 0.01 | 1.00 |
| Lymphnode dissection | 0.35 | 0.23 | 0.41 | 0.24 | 0.12 | 0.94 |  | 0.38 | 0.24 | 0.38 | 0.24 | 0.00 | 1.00 |
| Average value of Std.diff, Var.Ratio |  |  |  |  | 0.20 | 0.93 |  |  |  |  |  | 0.01 | 1.02 |
|  |  |  |  |  |  |  |  |  |  |  |  |  |  |

Abbreviations: ASA, American Society of Anesthesiologists; GNRI, Geriatric Nutritional Risk Index; IPTW, inverse-probability-of-treatment weighting; pStage, pathological stage.

**Supplementary Table 3a**	Median survival time analysis (unadjusted)

|  | Time at risk | Incidence rate | N | Survival time | | |
| --- | --- | --- | --- | --- | --- | --- |
|  |  |  |  | 25% | **50%** | 75% |
| Cohort 1 |  |  |  |  |  |  |
| GNRI cutoff value, 98 |  |  |  |  |  |  |
| <98 | 110,529 | 0.0007962 | 189 | 308 | **924** | 1878 |
| ≥98 | 25,897 | 0.0004634 | 36 | 797 | **1206** | 1436 |
| Total | 136,426 | 0.000733 | 225 | 446 | 976 | 1878 |
|  |  |  |  |  |  |  |
| Cohort 2 |  |  |  |  |  |  |
| GNRI cutoff value, 86 |  |  |  |  |  |  |
| <86 | 53,602 | 0.0010261 | 113 | 213 | **700** | 1423 |
| ≥86 | 82,824 | 0.0005433 | 112 | 723 | **1283** | 2115 |
| Total | 136,426 | 0.000733 | 225 | 446 | 976 | 1878 |

Abbreviations: GNRI, Geriatric Nutritional Risk Index.

**Supplementary Table 3b**	Median survival time analysis (after stabilized IPTW adjustment)

|  | Time at risk | Incidence rate | N | Survival time | | |
| --- | --- | --- | --- | --- | --- | --- |
|  |  |  |  | 25% | **50%** | 75% |
| Cohort 1 |  |  |  |  |  |  |
| GNRI cutoff value, 98 |  |  |  |  |  |  |
| <98 | 110,857 | 0.0007883 | 188.5957 | 308 | **924** | 1878 |
| ≥98 | 27,979 | 0.0004946 | 37.35374 | 930 | **1206** | 1436 |
| Total | 138,836 | 0.0007291 | 225.9494 | 446 | 976 | 1734 |
|  |  |  |  |  |  |  |
| Cohort 2 |  |  |  |  |  |  |
| GNRI cutoff value, 86 |  |  |  |  |  |  |
| <86 | 52,549 | 0.0010069 | 112.748 | 261 | **700** | 1423 |
| ≥86 | 85,010 | 0.0005367 | 111.8744 | 723 | **1283** | 2123 |
| Total | 137,559 | 0.0007164 | 224.6224 | 446 | 976 | 1878 |

Abbreviations: GNRI, Geriatric Nutritional Risk Index; IPTW, inverse-probability-of-treatment weight.
