# Supplementary material for: The Geriatric Nutritional Risk Index Predicts Short‐ and Long‐Term Outcomes in the Oldest‐Old With Colorectal Cancer: A Multi‐Institutional Analysis of 225 Nonagenarians
Source: Ann Gastroenterol Surg. 2026 Sep 29:10.1002/ags3.70288. Online ahead of print. doi: 10.1002/ags3.70288 (PMC13621199; doi:10.1002/ags3.70288)
Supplement: Supplementary file 2 [file AGS3-9999-0-s002.docx]

**Supplementary Figure 1**

**Supplementary Figure 2**
